# Hsa_circRNA_0088036 acts as a ceRNA to promote bladder cancer progression by sponging miR-140-3p

**DOI:** 10.1038/s41419-022-04732-w

**Published:** 2022-04-08

**Authors:** Jun Yang, Manlong Qi, Xiang Fei, Xia Wang, Kefeng Wang

**Affiliations:** 1 Department of Gastroenterology, Shengjing Hospital of China Medical University, Shenyang, 110004 China; 2 Department of Clinical Genetics, Shengjing Hospital of China Medical University, Shenyang, 110004 China; 3 Department of Urology, Shengjing Hospital of China Medical University, Shenyang, 110004 China

**Keywords:** Bladder cancer, Long non-coding RNAs

## Abstract

Circular RNAs (circRNAs) are a class of non-coding RNAs that play vital roles in cancer biology. However, the potential role of hsa_circRNA_0088036 in bladder cancer (BCa) remains unknown. Hsa_circRNA_0088036 was identified by microarray analysis and validated by quantitative real-time polymerase chain reaction. Functional assays were conducted to confirm the effects of hsa_circRNA_0088036 on the growth, migration, invasion, tumorigenesis, and metastasis of BCa cells. The luciferase reporter assay and RNA pull down assay were performed to investigate the interactions between hsa_circRNA_0088036, miR-140-3p, and forkhead box protein Q1 (FOXQ1). Upregulated expression of hsa_circRNA_0088036 in BCa tissues and cell lines was positively correlated with overall survival and clinicopathologic characteristics. Knockdown of hsa_circRNA_0088036 inhibited the growth, migration, and invasion of BCa cells both in vivo and in vitro. Mechanistically, hsa_circRNA_0088036 could directly interact with miR-140-3p and act as a miRNA sponge to modulate FOXQ1 expression. Knockdown of hsa_circRNA_0088036 inhibited the proliferation, migration, and metastasis of BCa cells via miR-140-3p/FOXQ1 signaling, suggesting that hsa_circRNA_0088036 is a potential biomarker and therapeutic target for BCa.

## Introduction

Bladder cancer (BCa) is the fourth most common type of cancer and the eighth leading cause of cancer-related death of men worldwide, and accounted for an estimated 81,400 new cases (4.5% of all new cancers) and 17,980 deaths (2.9% of all deaths) of men in the United States in 2020 [1]. BCa is a type of urological malignant tumor that originates from urothelial cells and generally progresses from non-muscle-invasive to muscle-invasive BCa [2]. Approximately one-third of patients are diagnosed with muscle-invasive BCa on the initial visit, with some cases having local or distant metastases [3, 4]. Surgery has little effect on advanced BCa with distant metastasis. Although some new chemotherapeutic agents and targeted therapies are still being tested in clinical trials, the development of new therapies against metastatic BCa is imperative.

Circular RNA (circRNA) is a class of covalently closed loop non-coding RNA (ncRNA) without 5’ to 3’ polarity [5]. CircRNAs are involved in many pathological and physiological processes, including the onset and progression of tumors [6, 7]. Tens of thousands of circRNAs are dysregulated in human tumors, as determined by high-throughput sequencing analyses [8]. Some circRNAs have been shown to affect the progression of BCa [9–11]. CircRNAs can function as protein-coding genes, RNA-binding protein sponges, and microRNA (miRNA) sponges. Revealing the impacts of circRNAs could help to better understand the molecular mechanisms underlying the carcinogenesis of BCa.

MiRNAs are also a class of ncRNAs that can negatively regulate gene expression by targeting the 3’-untranslated region (3’-UTR). Previous studies have reported that circRNAs can serve as miRNA sponges to inhibit translation of target mRNA [12, 13]. At present, the role of the circRNA-microRNA axis in BCa remains largely unknown, and thus, further investigations are needed.

In this study, a microarray was used to analyze circRNA expression profiles in BCa tissues. Hsa_circRNA_0088036 expression was remarkably upregulated in BCa tissues and promoted the proliferation, migration, and invasion of BCa cells. Hsa_circRNA_0088036 was found to upregulate FOXQ1 expression through miR-140-3p in BCa cells. The study results showed that hsa_circRNA_0088036 can bind to miR-140-3p by acting as a competitive endogenous RNA and modulate FOXQ1 expression, suggesting that hsa_circRNA_0088036 could be used as a biomarker and potential therapeutic target for BCa patients.

## Results

### Hsa_circRNA_0088036 expression is upregulated in BCa tissues and cell lines

**Fig. 1:**
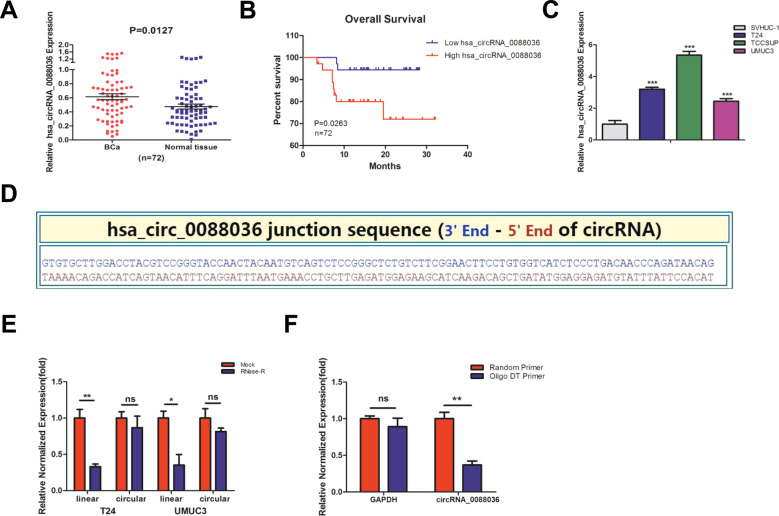
The expression of hsa_circRNA_0088036, which has a closed-looped structure, is upregulated in BCa tissues and cell lines. **A** Hsa_circRNA_0088036 expression in 72 paired human BCa and adjacent normal bladder tissues. **B** OS curves of BCa patients were analyzed according to hsa_circRNA_0088036 expression. **C** Hsa_circRNA_0088036 expression in SV-HUC-1 urothelial cells and T24, TCCSUP, and UMUC3 BCa cells. **D** Sequence of hsa_circRNA_0088036 in circBase. **E** Hsa_circRNA_0088036 was confirmed as a circRNA that is resistant to RNase R treatment. **F** The qRT-PCR analysis of poly(A)-tailed mRNAs revealed that non-poly(A)-tailed RNA hsa_circRNA_0088036 could not be reverse transcribed into cDNA with oligo DT primers.

Our previous study found that miR-140-3p suppressed the proliferation and invasion of BCa cells by targeting FOXQ1 expression [14]. Recent studies indicate that some ncRNAs regulate the expression patterns and functions of miRNAs, including circRNAs [15]. Hence, it is reasonable to speculate that some circRNAs could regulate the proliferation of BCa cells by interacting with miR-140-3p. Therefore, the circRNA expression profiles in BCa and adjacent normal bladder tissues were detected by microarray analysis (Supplementary Material 1). Data mining with miRanda and TargetScan predicted that hsa_circRNA_0088036 was the target of miR-140-3p. Therefore, hsa_circRNA_0088036 was chosen as a candidate circRNA, as the expression level was 6.97-fold greater in BCa tissues than adjacent normal bladder tissues, as determined by microarray analysis. Then, qRT-PCR analysis was conducted to detect the expression levels of hsa_circRNA_0088036 in 72 paired BCa and adjacent normal bladder tissues. As shown in Fig. 1A, hsa_circRNA_0088036 expression was higher in BCa tissues than adjacent normal bladder tissues. Meanwhile, high expression of hsa_circRNA_0088036 was significantly correlated with overall survival (OS) (Fig. 1B). Also, hsa_circRNA_0088036 expression was higher in T24, TCCSUP, and UMUC3 BCa cells, as compared to SV-HUC-1 urothelial cells (Fig. 1C).

### The relationship between hsa_circRNA_0088036 and the clinicopathologic characteristics of BCa

The relationship between hsa_circRNA_0088036 expression and the clinicopathologic characteristics of BCa was further explored. As shown in Table 1, high expression of hsa_circRNA_0088036 was significantly correlated with large tumor size, poor histological grade, and greater extents of lymphatic and distant metastases, but not age, sex, or tumor number. These data demonstrate that hsa_circRNA_0088036 could influence the growth and invasion of BCa cells.

**Table 1:** The relationship between hsa_circRNA_0088036 expression and clinicopathologic characteristics of Bca.

Parameters	Total	Low hsa_circRNA_0088036	High hsa_circRNA_0088036	*P*-value
All cases	72	23	49	
Age (years)				0.7993
≤ 60	41	14	27	
> 60	31	9	22	
Gender				0.4376
Male	44	16	28	
Female	28	7	21	
Tumor size (cm)				0.0220
≤ 3	32	15	17	
> 3	40	8	32	
Tumor number				0.3826
Solitary	55	16	39	
Multiple	17	7	10	
Histological grade				0.0258
Low	51	12	39	
High	21	11	10	
T-stage				0.0309
Ta-T1	50	20	30	
T2-T4	22	3	19	
Lymph node metastasis				0.0383
N0	47	19	28	
N1-N3	25	4	21	
Distant metastasis				0.0221
M0	52	21	31	
M1	20	2	18	

### Confirmation of the circular structure of hsa_circRNA_0088036

Bioinformatic analyses with the use of the circBase (http://www.circbase.org/) and CircInteractome (https://circinteractome.nia.nih.gov/) databases revealed that hsa_circRNA_0088036 was derived from exons 11 and 12 of the sushi domain containing 1 (SUSD1) locus located on chromosome 9q31.3-q32 (Fig. 1D). The genomic length of hsa_circRNA_0088036 was 1628 nt (Supplementary Fig. S1A), while the spliced sequence length was 279 nt (Supplementary Fig. S1B). Then, the circular structure of hsa_circRNA_0088036 was confirmed using the RNase R-digest assay. The results revealed that hsa_circRNA_0088036 was more resistant to the RNase R enzyme as compared to linear RNAs in T24 and UMUC3 cells (Fig. 1E). Similarly, oligo DT or random primers were used to verify the lack of a poly(A) tail of hsa_circRNA_0088036. As expected, GAPDH and poly(A) tail mRNAs could be reverse transcribed into cDNA with either the oligo DT or random primer, while hsa_circRNA_0088036 could only be reverse transcribed with a random primer (Fig. 1F). These results indicate that hsa_circRNA_0088036 has a closed-looped structure.

### Knockdown of hsa_circRNA_0088036 inhibits the proliferation, migration, and invasion of BCa cells

**Fig. 2:**
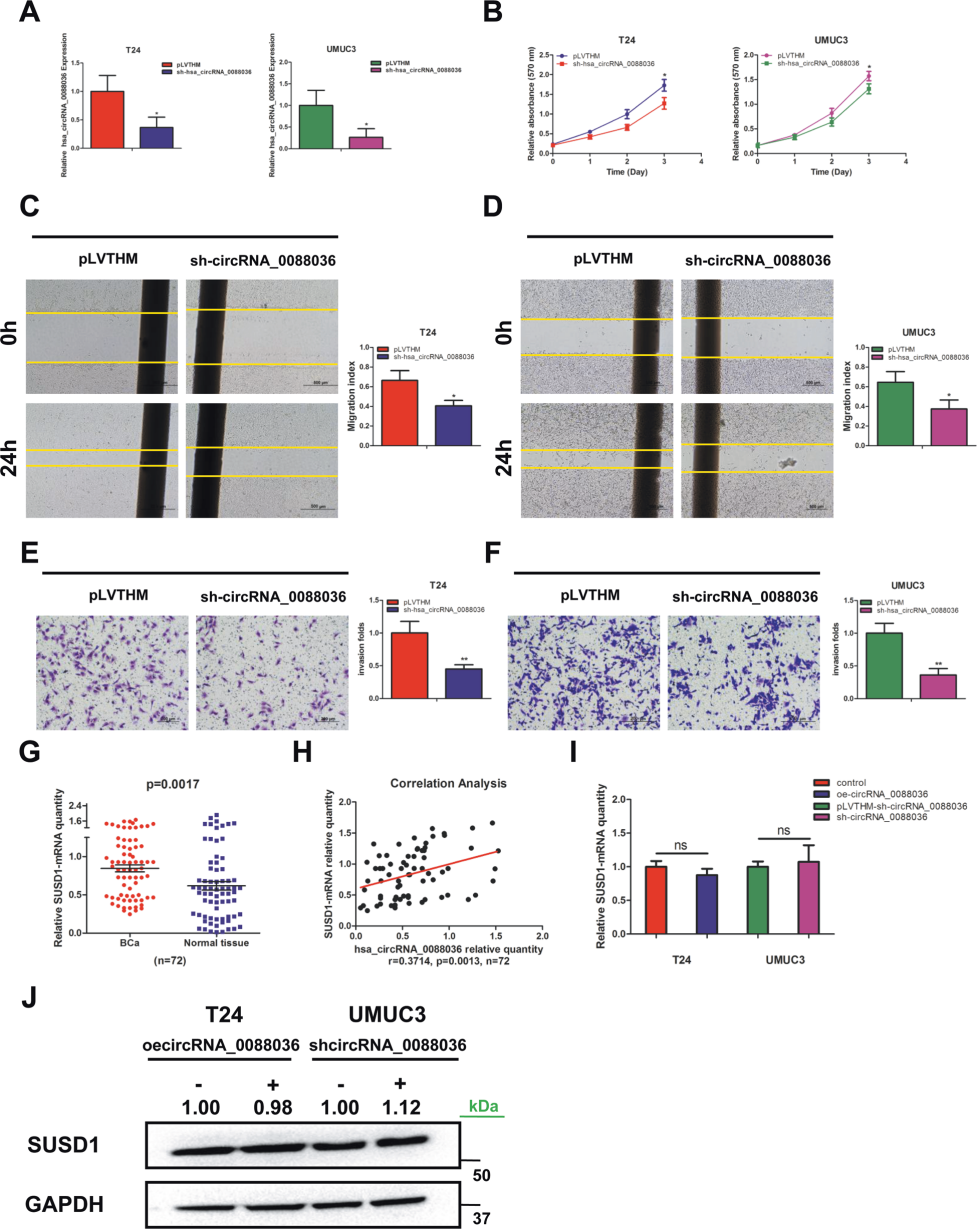
Knockdown of hsa_circRNA_0088036 inhibits the proliferation, migration, and invasion of BCa cells. **A** Verification of hsa_circRNA_0088036 knockdown by qRT-PCR analysis. **B** T24 and UMUC3 cells were transfected with hsa_circRNA_0088036-shRNA. Cell growth was measured with a cell proliferation assay. **C**, **D** T24 cells (**C**) and UMUC3 cells (**D**) were transfected with hsa_circRNA_0088036-shRNA. Cell migration was measured with the wound healing assay. **E**, **F** T24 cells (**E**) and UMUC3 cells (**F**) were transfected with hsa_circRNA_0088036-shRNA. 100 μL matrigel was added into upper chambers. Transwell invasion assays were performed. The invading cells were counted in 10 randomly chosen microscopic fields (100×) of each experiment and pooled. **G** SUSD1 expression in 72 paired human BCa and adjacent normal bladder tissues. **H** Correlation analysis of SUSD1 mRNA and hsa_circRNA_0088036 level was performed with the Pearson’s correlation coefficient. **I** The qRT-PCR analysis was performed to detect the expression of SUSD1 mRNA after overexpression of hsa_circRNA_0088036 in T24 cells and knockdown of hsa_circRNA_0088036 in UMUC3 cells. **J** Western blot analysis was performed to detect the expression of SUSD1 after overexpression of hsa_circRNA_0088036 in T24 cells and knockdown of hsa_circRNA_0088036 in UMUC3 cells.

The effects of hsa_circRNA_0088036 on the proliferation of BCa cells were investigated. First, hsa_circRNA_0088036 was knocked down in T24 and UMUC3 cells using backsplice junction-specific siRNA. The qRT-PCR results verified that the expression of hsa_circRNA_0088036 was effectively down-regulated in T24 and UMUC3 cells (Fig. 2A). The cell proliferation assay results revealed that knockdown of hsa_circRNA_0088036 significantly inhibited the growth of BCa cells (Fig. 2B). Similar results were obtained for both T24 and UMUC3 cells with the wound healing assay (Fig. 2C, D) and transwell invasion assay (Fig. 2E, F).

Together, the results presented in Fig. 2A–F demonstrate that knockdown of hsa_circRNA_0088036 significantly suppressed the proliferation, migration, and invasion of BCa cells.

### Hsa_circRNA_0088036 has no influence on its linear transcript

Recent studies revealed that circRNAs can modulate the expression of their corresponding linear transcripts [16, 17]. Therefore, the relationship between hsa_circRNA_0088036 and its linear transcripts (SUSD1) was investigated. The results of qRT-PCR analysis showed that SUSD1 expression was higher in BCa tissues than adjacent normal bladder tissues (Fig. 2G). A positive correlation between hsa_circRNA_0088036 and SUSD1 was revealed by the Pearson’s correlation coefficient (Fig. 2H). However, artificial modification of hsa_circRNA_0088036 expression had no effect on the mRNA and protein levels of SUSD1 (Fig. 2I, J, and Supplementary Material 2A, B). Together, these results suggest that SUSD1 is unlikely the target of hsa_circRNA_0088036.

### Hsa_circRNA_0088036 upregulates FOXQ1 expression through miR-140-3p in BCa cells

The results of our previous study indicated that miR-140-3p can inhibit the proliferation and invasion of BCa cells by suppression of FOXQ1 expression and microarray analysis and data mining showed that hsa_circRNA_0088036 might interact with miR-140-3p [14]. Therefore, further analysis was conducted to determine whether FOXQ1 is a downstream target of hsa_circRNA_0088036.

**Fig. 3:**
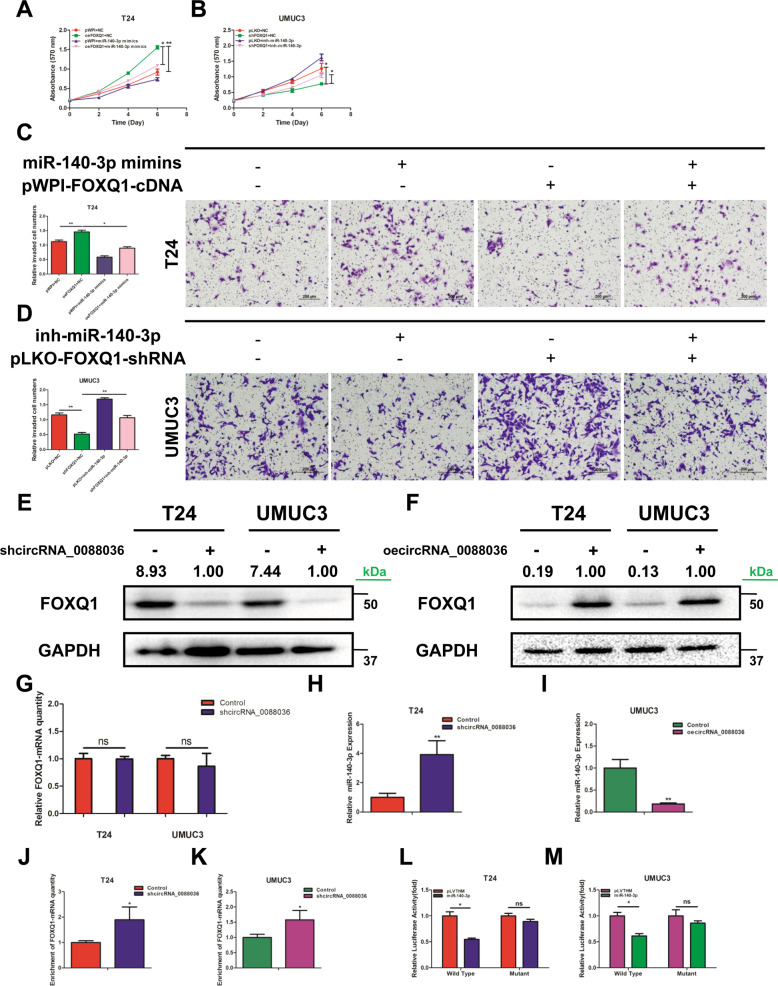
Hsa_circRNA_0088036 upregulates FOXQ1 expression through miR-140-3p in BCa cells. **A** The addition of miR-140-3p mimics partially rescued the growth of UMUC3 cells transfected with functional FOXQ1-cDNA, as determined by the cell proliferation assay. **B** The addition of a miR-140-3p inhibitor partially rescued the growth of UMUC3 cells transfected with FOXQ1-shRNA, as determined by the cell proliferation assay. **C** Transwell invasion assays were performed using four groups of T24 cells (pWPI+NC, oeFOXQ1, oemiR-140-3p, and oeFOXQ1+oemiR-140-3p). 100 μL matrigel was added into upper chambers. The invading cells were counted in 10 randomly chosen microscopic fields (100×) of each experiment and pooled. **D** Transwell invasion assays were performed using four groups of UMUC3 cells (pLKO+NC, shFOXQ1, shmiR-140-3p, and shFOXQ1+shmiR-140-3p). 100 μL matrigel was added into upper chambers. The invading cells were counted in 10 randomly chosen microscopic fields (100×) of each experiment and pooled. **(E**, **F)** Western blot analysis was performed to detect the expression of FOXQ1 after knockdown (**E**) and overexpression (**F**) of hsa_circRNA_0088036 in T24 and UMUC3 cells. **G** The qRT-PCR analysis was performed to detect the expression of FOXQ1 mRNA after knockdown of hsa_circRNA_0088036 in T24 and UMUC3 cells. **H**, **I** The qRT-PCR analysis was performed to detect the expression of miR-140-3p after knockdown of hsa_circRNA_0088036 in T24 cells (**H**) and overexpression of hsa_circRNA_0088036 in UMUC3 cells (**I**). **J**, **K** An argonaute2 immunoprecipitation assay was performed after knockdown of hsa_circRNA_0088036 in T24 (**J**) and UMUC3 (**K**) cells. **L**, **M** Co-transfection of wild type or mutant seed regions of FOXQ1 3’-UTR constructs with miR-140-3p in T24 (**L**) and UMUC3 cells (**M**). The luciferase assay is applied to detect luciferase activity.

First, the role of miR-140-3p/FOXQ1 in the proliferation and invasion of BCa cells was verified again by rescue experiments in T24 and UMUC3 cells. MTT results revealed that miR-140-3p mimics could partially reverse the elevation mediated by upregulation of FOXQ1 in T24 cells (Fig. 3A). A miR-140-3p inhibitor could partially reverse the inhibiting effect mediated by downregulation of FOXQ1 in UMUC3 cells (Fig. 3B). Similar results were obtained in both T24 and UMUC3 cells by cell invasion assays (Fig. 3C, D). The above data demonstrated that miR-140-3p inhibited FOXQ1-induced pathogenesis of BCa cells.

Second, the results of western blot analysis indicated that knockdown of hsa_circRNA_0088036 decreased the expression of FOXQ1 as compared to the control in both T24 and UMUC3 cells (Fig. 3E and Supplementary Material 3A, B). In contrast, upregulation of hsa_circRNA_0088036 increased FOXQ1 expression (Fig. 3F and Supplementary Material 3C, D). To confirm whether hsa_circRNA_0088036 regulates FOXQ1 expression through miR-140-3p, hsa_circRNA_0088036 was knocked down and FOXQ1 expression was detected in T24 and UMUC3 cells. The qRT-PCR results showed only slight changes in FOXQ1 mRNA levels as compared to the control (Fig. 3G). The mismatch phenomenon of mRNA and protein levels implies that hsa_circRNA_0088036 might indirectly regulate FOXQ1 expression, which confirms our hypothesis that hsa_circRNA_0088036 might regulate FOXQ1 expression through miR-140-3p at the post-transcriptional level.

Third, hsa_circRNA_0088036 was manipulated and miR-140-3p expression was detected. The qRT-PCR results revealed that miR-140-3p expression increased after knockdown of hsa_circRNA_0088036 in T24 cells. In contrast, upregulation of hsa_circRNA_0088036 decreased miR-140-3p expression in UMUC3 cells (Fig. 3H, I).

Finally, the results of the AGO2 assay showed that knockdown of hsa_circRNA_0088036 enhanced FOXQ1 mRNA levels in T24 and UMUC3 cells (Fig. 3J, K), which confirmed that miR-140-3p was part of the RNA-induced silencing complex to inhibit FOXQ1 mRNA degradation and translation. The results of a luciferase reporter assay was conducted again to verify that miR-140-3p modulated FOXQ1 expression by binding to the mRNA 3’-UTR (Fig. 3L, M).

Together, the results presented in Fig. 3A–M indicate that hsa_circRNA_0088036 can modulate FOXQ1 expression via miR-140-3p by binding to the 3’-UTR of FOXQ1 mRNA.

### Hsa_circRNA_0088036 functions as a sponge for miR-140-3p

**Fig. 4:**
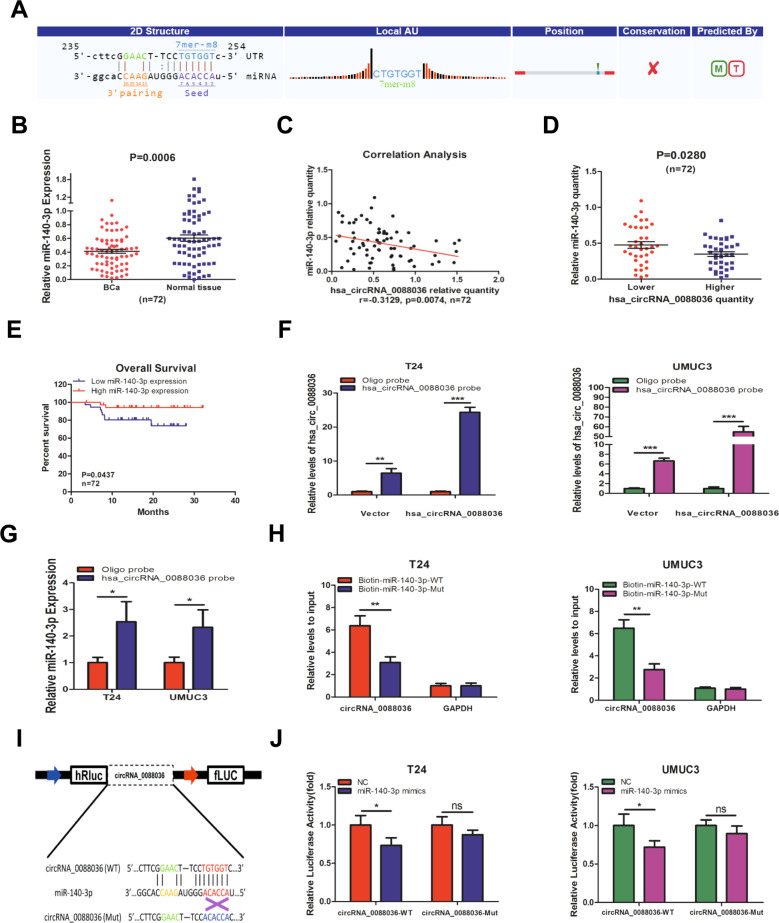
Hsa_circRNA_0088036 functions as a sponge for miR-140-3p. **A** Potential binding sites of hsa_circRNA_0088036 and miR-140-3p. **B** MiR-140-3p expression in 72 paired human BCa and adjacent normal bladder tissues. **C** Correlation analysis of miR-140-3p and hsa_circRNA_0088036 was performed with the Pearson’s correlation coefficient. **D** MiR-140-3p levels were measured in patients with low hsa_circRNA_0088036 (*n* = 36) and high hsa_circRNA_0088036 (*n* = 36) expression. **E** OS curves of BCa patients were analyzed according to miR-140-3p expression. **F** Relative levels of hsa_circRNA_0088036 in T24 and UMUC3 lysates after RNA pull down using oligo probe or hsa_circRNA_0088036 probe. **G** Dual luciferase reporter assays demonstrated that miR-140-3p is a direct target of hsa_circRNA_0088036. **H** An immunoprecipitation assay was performed with primers specific for hsa_circRNA_0088036. **I** A schematic of hsa_circRNA_0088036 WT and MUT luciferase reporter vectors. **J** Relative luciferase activities were calculated in cells co-transfected with miR-140-3p mimics and NC and WT or MUT luciferase reporter vectors.

Recent studies have shown that circRNAs can interact with miRNAs as miRNA sponges to modulate the expression of downstream genes [18]. Thus, whether and how hsa_circRNA_0088036 interacts with miR-140-3p was investigated. Bioinformatics software (Target and miRanda) was used to identify the binding sites between hsa_circRNA_0088036 and miR-140-3p (Fig. 4A). The qRT-PCR results revealed that miR-140-3p expression was lower in BCa tissues than adjacent normal bladder tissues (Fig. 4B). There was also a negative correlation (Pearson *r* = −0.3129, *p* = 0.0074) between hsa_circRNA_0088036 and miR-140-3p in 72 paired BCa tissues (Fig. 4C), suggesting that lower hsa_circRNA_0088036 expression was associated with higher miR-140-3p expression (Fig. 4D). OS of BCa patients in the high miR-140-3p expression group was significantly higher than that of patients in the low miR-140-3p expression group (Fig. 4E). These results suggest that miR-140-3p might be a tumor suppressor of BCa.

An RNA pull down assay was conducted using a biotinylated hsa_circRNA_0088036 probe. The qRT-PCR was performed to confirm the pulldown effect in T24 and UMUC3 cells (Fig. 4F). The results showed that miR-140-3p could be pulled down by hsa_circRNA_0088036 in T24 and UMUC3 cells (Fig. 4G). Subsequently, a biotin-coupled miRNA capture assay was performed to detect the sponge effect between hsa_circRNA_0088036 and miR-140-3p. BCa cells transfected with oehsa_circRNA_0088036 were also transfected with WT or mutant (MUT) biotin-labeled miR-140-3p. The results revealed that the abundance of hsa_circRNA_0088036 was higher in the biotin-miR-140-3p-WT group than the biotin-miR-140-3p-MUT group (Fig. 4H). Then, BCa cells were transfected with WT or MUT hsa_circRNA_0088036 fragments followed by miR-140-3p mimics or a negative control (NC). The results showed that the luciferase activity of hsa_circRNA_0088036-WT decreased in the miR-140-3p mimic group as compared to the NC group, but not that of hsa_circRNA_0088036-MUT in T24 and UMUC3 cells (Fig. 4I, J).

Together, the results presented in Fig. 4A–J demonstrate that hsa_circRNA_0088036 could function as a sponge for miR-140-3p.

### Hsa_circRNA_0088036 promotes the proliferation, migration, and invasion of BCa cells by altering miR-140-3p-FOXQ1 signals

**Fig. 5:**
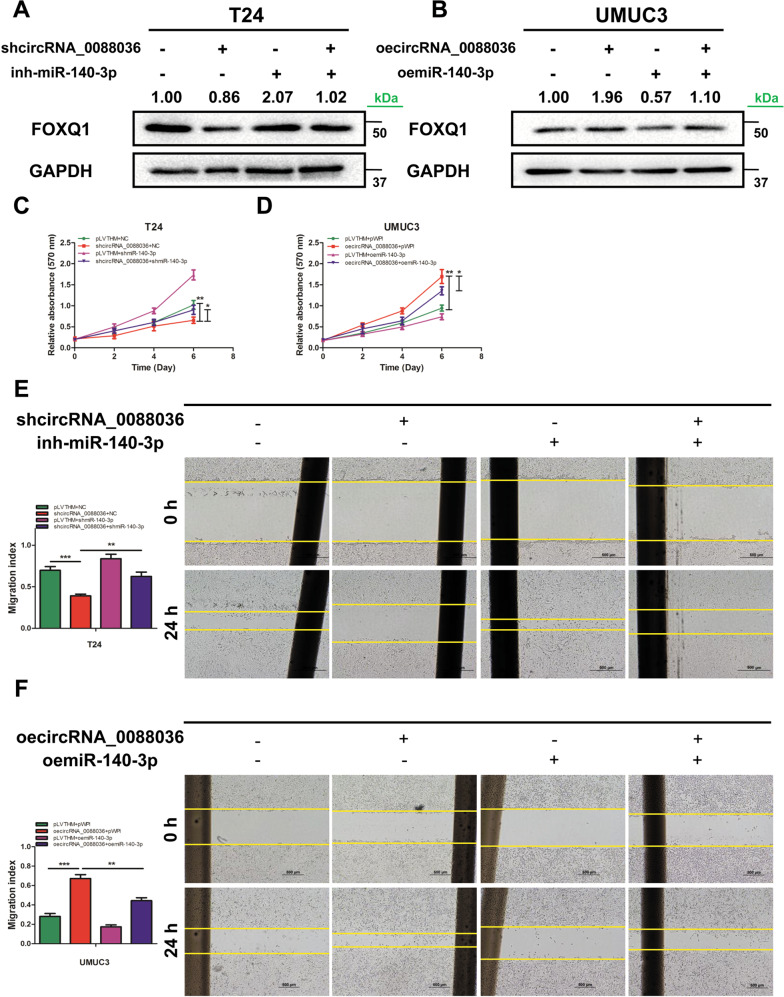

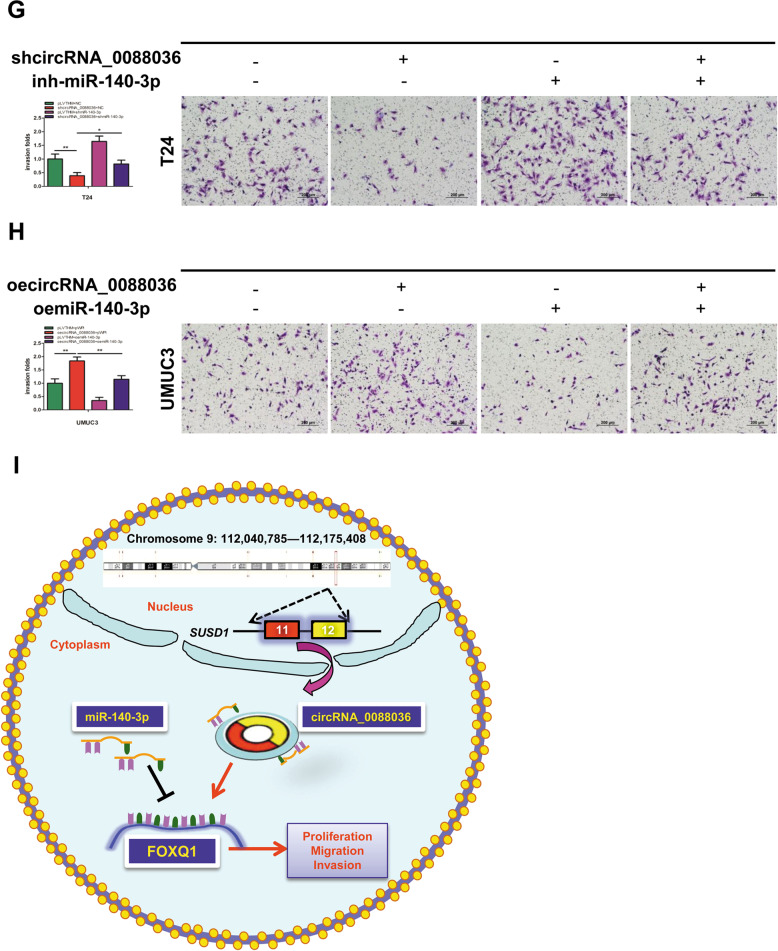
Hsa_circRNA_0088036 promotes the proliferation, migration, and invasion of BCa cells by altering miR-140-3p-FOXQ1 signals. **A** Protein levels of FOXQ1 in T24 cells were determined by western blot analysis after co-transfection with shhsa_circRNA_0088036 and an miR-140-3p inhibitor. **B** Protein levels of FOXQ1 in UMUC3 cells were determined by western blot analysis after co-transfection with oehsa_circRNA_0088036 and miR-140-3p mimics. **C** The addition of an miR-140-3p inhibitor partially rescued the growth of T24 cells transfected with shhsa_circRNA_0088036, as determined by the cell proliferation assay. **D** The addition of miR-140-3p mimics partially rescued the growth of UMUC3 cells transfected with oehsa_circRNA_0088036, as determined by the cell proliferation assay. **E** Wound healing assays were performed using four groups of T24 cells (pLVTHM+NC, shhsa_circRNA_0088036, shmiR-140-3p, and shhsa_circRNA_0088036 + shmiR-140-3p). **F** Wound healing assays were performed using four groups of UMUC3 cells (pLVTHM+pWPI, oehsa_circRNA_0088036, oemiR-140-3p, and oehsa_circRNA_0088036 + oemiR-140-3p). **G** Transwell invasion assays were performed using four groups of T24 cells (pLVTHM+NC, shhsa_circRNA_0088036, shmiR-140-3p, and shhsa_circRNA_0088036 + shmiR-140-3p). 100 μL matrigel was added into upper chambers. The invading cells were counted in 10 randomly chosen microscopic fields (100×) of each experiment and pooled. **H** Transwell invasion assays were performed using four groups of UMUC3 cells (pLVTHM+pWPI, oehsa_circRNA_0088036, oemiR-140-3p, and oehsa_circRNA_0088036 + oemiR-140-3p). 100 μL matrigel was added into upper chambers. The invading cells were counted in 10 randomly chosen microscopic fields (100×) of each experiment and pooled. **I** A schematic diagram indicating that hsa_circRNA_0088036 promoted the proliferation of BCa cells through miR-140-3p/FOXQ1 signaling.

Next, the influence of hsa_circRNA_0088036 on the proliferation, migration, and invasion of BCa cells was explored by altering miR-140-3p-FOXQ1 signals. The western blot results indicated that downregulation of hsa_circRNA_0088036 decreased the expression of FOXQ1 in T24 cells, which was partially reversed by the addition of a miR-140-3p inhibitor (Fig. 5A and Supplementary Material 4A, B). As expected, overexpression of miR-140-3p partially reversed oehsa_circRNA_0088036-enhanced FOXQ1 expression in UMUC3 cells (Fig. 5B and Supplementary Material 4C, D).

The cell proliferation assay results showed that the miR-140-3p inhibitor partially reversed shhsa_circRNA_0088036-induced suppression of the growth of T24 cells (Fig. 5C). On the other hand, overexpression of miR-140-3p partially reversed oehsa_circRNA_0088036-induced growth of UMUC3 cells (Fig. 5D).

The results of the wound healing assay (Fig. 5E, F) and transwell invasion assay (Fig. 5G, H) were similar for both the T24 and UMUC3 cells.

Taken together, these data illustrate that hsa_circRNA_0088036 can promote the proliferation, migration, and invasion of BCa cells by altering miR-140-3p-FOXQ1 signaling (Fig. 5I).

### Knockdown of hsa_circRNA_0088036 inhibits BCa cell growth and metastasis in vivo

**Fig. 6:**
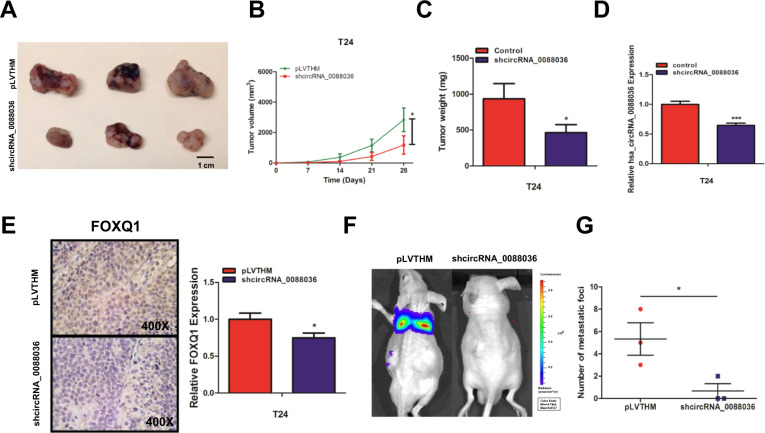
Knockdown of hsa_circRNA_0088036 inhibited the growth of BCa cells in vivo. **A** Representative images of xenograft tumors in six nude mice. **B**, **C** Tumor volume (**B**) and weight (**C**) were dramatically decreased in the shhsa_circRNA_0088036 group. **D** Knockdown of hsa_circRNA_0088036 was confirmed in xenograft tumor models. **E** Immunohistochemical staining of FOXQ1 in xenografts. **F** Representative bioluminescence images of mice metastasis model. **G** Statistical analysis of metastasis foci.

To explore the effect of hsa_circRNA_0088036 on the growth and metastasis of BCa cells in vivo, T24 cells were transfected with shhsa_circRNA_0088036 or the NC. Then, the cells were subcutaneously injected into nude mice. The tumor volume and weight were dramatically decreased in the shhsa_circRNA_0088036 group as compared to the NC group at 4 weeks post-injection (Fig. 6A–C). The qRT-PCR was performed to detect hsa_circRNA_0088036 expression in xenografts (Fig. 6D). As expected, FOXQ1 expression was decreased in the shhsa_circRNA_0088036 group, as determined by immunohistochemical analysis (Fig. 6E). Bioluminescence imaging indicated that knockdown of hsa_circRNA_0088036 reduced tumor metastasis in vivo (Fig. 6F). All three mice in the NC group developed metastases, while only one of the three mice in the shhsa_circRNA_0088036 group occurred. Hsa_circRNA_0088036 knockdown cells formed fewer metastatic foci in lungs than the NC group (Fig. 6G).

## Discussion

High-throughput sequencing has been used to identify numerous circRNAs, including many that act as suppressors or oncogenes in human cancers [19]. However, the distinctive functions of circRNAs in BCa remain unknown, and thus, further investigations are needed. Here, we identified a new circRNA, hsa_circRNA_0088036 derived from exons 11 and 12 of the SUSD1 gene. Our data revealed that hsa_circRNA_0088036 was upregulated in 72 BCa tissues and cell lines, suggesting its oncogenic effect. Downregulation of hsa_circRNA_0088036 was found to suppress the proliferation, migration, and invasion of BCa cells, whereas overexpression had opposite effects. Upregulation of hsa_circRNA_0088036 was significantly correlated with large tumor size, poor histological grade, and greater extents of lymphatic and distant metastases.

Upregulation of hsa_circRNA_0088036 increased FOXQ1 expression, implying a potential regulatory network between hsa_circRNA_0088036 and FOXQ1. Notably, recent studies have demonstrated that circRNAs can function as miRNA sponges to modulate downstream endogenous target genes [13, 20]. Most of these miRNA sponges are derived from one or more exons of known protein-coding genes by back-splicing [12]. CiRS-7/CDR1as was the first identified miRNA sponge that negatively regulates miR-7 in many human cancers [12, 13, 21–25]. Circ-ITCH can also function as a miRNA sponge to protect target genes from repression in human cancers of the cervix [26], breast [27], ovary [28], bladder [10], and papillary thyroid [29]. In this study, the results of the biotinylated RNA pull-down and luciferase reporter assays showed that hsa_circRNA_0088036 interacts with miR-140-3p in BCa cells. Subsequently, hsa_circRNA_0088036 was found to play an oncogenic role in BCa cells. Furthermore, overexpression of miR-140-3p antagonized hsa_circRNA_0088036-mediated enhancement of the growth, migration, and invasion of BCa cells. Taken together, these findings indicate that hsa_circRNA_0088036 can function as a sponge of miR-140-3p and inhibition of hsa_circRNA_0088036 expression might be used as a novel method to inhibit the proliferation of BCa cells.

Accumulating studies have implied that miRNAs can reduce the expression of target genes by binding to the 3’-UTR of target mRNAs at the post-transcriptional level, resulting in mRNA degradation or translation depression [30]. The results of our previous study revealed that miR-140-3p inhibited the proliferation and invasion of BCa cells by reducing FOXQ1 expression. The results of the present study verified that hsa_circRNA_0088036 upregulated FOXQ1 expression via miR-140-3p in BCa cells. FOXQ1 is known to act as an oncogene in many cancers [31–41], including BCa [42]. Jiao et al. [42] found that lncRNA MALAT1 increased FOXQ1 expression to promote cell proliferation and metastasis through sponging miR-124 in BCa cells. However, it remains unknown whether hsa_circRNA_0088036 regulates the proliferation of BCa cells by modulating miR-140-3p/FOXQ1 signals. The results of the present study verified that overexpression of hsa_circRNA_0088036 increased the expression of FOXQ1, as well as the proliferation, migration, and invasion in BCa cells, while miR-140-3p mimics antagonized hsa_circRNA_0088036-mediated enhancement of FOXQ1 expression and the proliferation of BCa cells. Hence, this study provides evidence that FOXQ1 is regulated by circRNA at the post-transcriptional level in BCa cells. Interestingly, our study found that miR-140-3p could inhibit FOXQ1 translation without the necessity for FOXQ1 mRNA degradation, which was different from the results of many other studies [43, 44]. The relative contributions of translational inhibition and mRNA degradation and whether these pathways act sequentially or in parallel remain controversial. There is now increasing evidence that translation inhibition of miRNA targets is the main event in this mechanism [45]. Several research groups have verified that translational inhibition can be performed without undergoing mRNA degradation [46–48]. Some miRNA-mRNA assays also get similar conclusions [49, 50]. Therefore, our study provides a new insight into the miRNA-mediated repression.

To date, numerous circRNAs have been identified and verified to play vital roles in tumorigenesis. However, there are still many problems to be solved. CircRNAs are considered as a class of ncRNAs, but recent studies revealed that some code for protein products [51, 52]. Therefore, further studies are needed on this new frontier in exploration of circRNAs in addition to miRNA sponges. New and effective methods and bioinformatic tools are needed to predict circRNA target genes. CircRNA-miRNA-mRNA regulatory networks must also be refined to reveal the pathogenetic mechanisms and search for novel diagnostic biomarkers. With the progression of research and technology, the functions of most circRNAs will be revealed and applied against human cancers.

Because of the conservatism, stability, specificity, and abundance in blood, saliva, and other body fluids, circRNAs can be detected clinically, indicating potential use as noninvasive biomarkers. In this study, hsa_circRNA_0088036 was positively correlated with tumor size, lymphatic metastasis, and distant metastasis. OS was poorer for patients with higher hsa_circRNA_008803. Therefore, hsa_circRNA_0088036 might serve as ideal and novel diagnostic and prognostic biomarkers in BCa.

In summary, hsa_circRNA_0088036 was dramatically upregulated in BCa and correlated with poor clinicopathologic outcomes. Mechanistically, knockdown of hsa_circRNA_0088036 suppressed the proliferation, migration, invasion, and metastasis of BCa cells by sponging miR-140-3p and enhanced the inhibition effect of miR-140-3p on FOXQ1 expression, suggesting that hsa_circRNA_0088036 could be a potential biomarker and therapeutic target for BCa.

## Materials and methods

### Patients and BCa samples

A total of 72 BCa samples and adjacent normal bladder tissues were obtained from the Department of Biobank of Shengjing Hospital of China Medical University. They all underwent radical cystectomy and were diagnosed as BCa by three pathologists. All the specimens were moved and snap-frozen in liquid nitrofen within 10 mins after removal from the body. The collection of these samples was authorized by the Ethics Committee of Medical Research and New Technology, Shengjing Hospital of China Medical University (Ref#2016PS449K). The ethics consents were acquired from patients before sample collection.

### Reagents

FOXQ1 antibody was purchased from Biorbyt Ltd (host: rabbit; catalog number: orb77456 for western blot and orb53843 for IHC). GAPDH antibody (0411) was purchased from Santa Cruz Biotechnology (host: mouse; catalog number: sc-47724). Anti-mouse/rabbit second antibodies were from Invitrogen (Grand Island, NY). The antibodies were kept at −20 °C.

### CircRNAs microarray hybridization and data analysis

Total RNA was treated with Rnase R (EpiCentre Inc., Madison, USA) to enrich circRNAs. The enriched circRNAs were amplified and transcribed into fluorescent cRNAs utilizing random primers according to Arraystar Super RNA Labeling protocol (ArraystarInc, Rockville, USA). The slides were incubated for 17 h at 65 °C in an Agilent Hybridization Oven. Scanned images were imported into Agilent Feature Extraction software for raw data extraction. When comparing two groups of profile differences, the “fold change” between two groups for each circRNA was computed. CircRNAs having fold changes ≥ 2 and *P*-values < 0.05 were selected as the significantly differentially expressed. Volcano Plots and Hierarchical Clustering were used to show the differentially expressed circRNAs.

### Cell culture and transfection

Human BCa cells (T24, UMUC3, and TCCSUP) and normal bladder epithelial cells (SV-HUC-1) were obtained from American Type Culture Collection (ATCC, Manassas, USA). All cell lines were cultured in DMEM (Invitrogen, Grand Island, USA) in a humidified atmosphere at 37 °C with 5% CO2. The cells were authenticated as mycoplasma and bacteria free following ATCC’s instructions. Small interfered RNAs (hsa_circRNA_0088036 siRNA: AACCCAGATAACAGTAAAACA), miR-140-3p mimics, miR-140-3p inhibitors, and their negative controls were transfected into BCa cells by lipofectamine 3000 (Invitrogen, Carlsbad, USA).

### RNA extraction and quantitative real-time polymerase chain reaction (qRT-PCR)

RNAs were extracted by Trizol reagent and reverse transcribed using Superscript III transcriptase (Invitrogen, Grand Island, USA). QRT-PCR was conducted by a Bio-Rad CFX96 system with SYBR green to measure the mRNA expression. MiRNAs were extracted by a PureLink® miRNA kit. GAPDH and U6 were used as internal standards. All the primers were expressed as follows: hsa_circRNA_0088036: F-primer: ACGTCCGGGTACCAACTACA, R-primer: TCCATCTCAAGCAGGTTTCA; FOXQ1: F-primer: CTACTCGTACATCGCGCTCA, R-primer: ACCTTGACGAAGCAG TCGTT. miR-140-3p: TACCACAGGGTAGAACCACGG.

### RNA extraction from formalin-fixed, paraffin-embedded (FFPE) blocks

The tissue slides were cut into 30 μM sections from the paraffin block using a microtome. Then, they were placed into siliconized tubes, and added 1 mL 100% xylene. After that, the tissue slides were incubated at 50 °C for 3 min to melt the paraffin, and were centrifuged for 2 min to pellet the tissue. The pellet was washed twice with 1 mL 100% ethanol, and air dried. Subsequently, 150 μL 1x protease K digestion buffer was added into each sample, and incubated at 55 °C for 3 h. 1 mL Trizol was added, and incubated at 30 °C for 5 min to dissociate nucleoprotein complexes. 0.2 mL chloroform was added, and incubated at 30 °C for 3 min after vortexing 15 sec. The samples were centrifuged at 12,000 for 15 min. Then, the aqueous phase was transferred to a fresh tube, and 10 μg glycogen was added and mixed. The total RNA was precipitated by 0.6 mL isopropyl alcohol, and centrifuged at 12,000 for 10 min. Finally, the RNA pellet was washed by 100% ethanol and dissolved in RNase-free water.

### RNase R digestion

The hsa_circRNA_0088036 from T24 and UMUC3 cells were treat with RNase R (3 U/μg, Epicenter Biotechnologies, Madison, USA). For control, 2 μg RNAs were mixed with DEPC water and 10x RNase R buffer; For RNase R digestion, 2 μg RNAs were mixed with RNase R and 10x RNase R buffer. Then, the two groups were incubated for 15 min at 37 °C. The treated RNAs were detected by qRT-PCR by specific primers. GAPDH was used as an internal control.

### Cell proliferation assay

Transfected cells were plated into 24-well plates (2 × 10^3^ cells per well). 50 µL MTT reagent (10 mg/mL) was added to each well at 1, 2, 3, and 4 day after inoculation. The medium was sucked out and 500 µL DMSO was added. 100 µL DMSO was transferred to 96-well plates and the absorbance was measured at 570 nM.

### Wound healing assay

Transfected cells were plated into 6-well plates. Wounds were manufactured by a 200 µL pipet tip. PBS was used three times to wash the cell debris, and then the cells were incubated with serum-free media. The wound was permitted to heal for 24 h. Cells were photographed at 0 h and 24 h using a phase-contrast microscope. Migration index was calculated after three times experiments using ImageJ software.

### Cell invasion assay

BCa cells were cultured in a 6-well plate and incubated for 72 h. 100 μL Matrigel (0.2 mg/mL, BD Corning, Corning, USA) was added into upper chambers and incubated at 37 °C for 2 h. 750 μL culture medium was added into lower chamber. Then, cells were collected and plated into upper chambers (1 × 10^5^/mL) and incubated at 37 °C for 18 h. The medium was removed, and cells were washed twice by PBS. After that, cells were permeabilized using methanol at RT for 20 min. Crystal violet (0.1%) was used to stain cells for 10 min. Non-invasion cells were scraped off with cotton swabs. Invasive cells were counted under a light microscope.

### Western blot assay

BCa cells are lysed in lysis buffer on ice and the protein (50 μg) was extracted and transferred onto PVDF membranes (Millipore, Billerica, USA). Then the membranes were blocked by bovine serum albumin (Sigma-Aldrich, St. Louis, MO) for 1 h at room temperature and bred with primary antibodies at 4 °C overnight. Thereafter, the membranes were incubated with secondary antibodies at room temperature for 1 h. The next day, anti-mouse or anti-rabbit IgG secondary antibodies were used for 1 h at the concentration of 1:5000 at room temperature and rinsed 10 min by TBST 3 times. The signals were visualized using a chemiluminescent detection system (Thermo Fisher Scientific, Shanghai, China).

### Biotin-coupled probe pull-down assay

The biotinylated hsa_circRNA_0088036 probe and oligo probe were incubated with streptavidin magnetic beads (Thermo Fisher Scientific, Shanghai, China) at room temperature for 2 h. About 1 × 10^7^ BCa cells were harvested, lysed and incubated with probe-coated beads at 4 °C overnight. The RNA complexes combining on the beads were extracted with RNeasy Mini Kit (QIAGEN, Shanghai, China) and evaluated by qRT-PCR assay.

### Biotin-coupled miRNA capture

BCa cells were transfected with biotinylated miRNA mimics or nonsense control (GenePharma, Shanghai, China) by lipofectamine 3000 (Invitrogen, Carlsbad, USA). The cells were harvested and lysed 24 h after transfection. Then, the cell lysates were incubated with streptavidin magnetic beads (Thermo Fisher Scientific, Shanghai, China) at 4 °C for 2 h. The beads were washed and the bound RNAs were extracted by RNeasy Mini Kit (QIAGEN, Shanghai, China). The abundance of hsa_circRNA_0088036 was analyzed by qRT-PCR analysis.

### Luciferase reporter assay

After seeding on 24-well plates, T24 and UMUC3 cells were co-transfected with target luciferase (with 1 ng/mL pRL-TK plasmid as internal control) and miR-140-3p mimics using lipofectamine 3000 transfection reagent (Invitrogen, Carlsbad, CA). 24 h after transfection, renilla and firefly luciferase activities were measured by dual-luciferase reporter assay system (Promaga, Madison, WI). Ratios of luminescence were calculated.

### RNA immunoprecipitation (RIP) assay

Magna RIP^TM^-Binding Protein Immunoprecipitation Kit (Millipore, Bedford, USA) was obtained to perform the RIP assay. BCa cells were lysed by lysis buffer with RNase inhibitors and proteinase. The RIP lysates were incubated with RIP buffers containing magnetic beads coupled with non-specific mouse IgG antibodies or human anti-AGO2 antibodies. Each protein immunoprecipitate was digested with protease K [53]. Hsa_circRNA_0088036 enrichment was detected by qRT-PCR assay.

### Immunohistochemistry (IHC)

The tissue slides were incubated with antibody against FOXQ1. The slides were treated with 1x EDTA 10 min for antigen retrieval, and incubated with endogenous peroxidase blocking solution and the primary antibody overnight. After rinsing with tris-buffered saline, the slides were incubated 45 min with biotin-conjugated secondary antibody, washed, and then incubated with enzyme conjugate horseradish peroxidase (HRP)-streptavidin. Freshly prepared DAB (Zymed, South San Francisco, CA) was used as a substrate to detect HRP. Finally, the slides were counter-stained with hematoxylin and mounted with aqueous mounting media. Positive cells were calculated as the number of immunopositive cells x 100% divided by total number of cells/field in 10 random fields at 400x magnification [54].

### Xenograft nude mouse model

Six six-week-old nude mice were purchased from Shanghai SLAC Laboratory Animal Co. Ltd (Shanghai, China). They were randomly divided into experimental and control groups. Approximately 1 × 10^6^ T24 cells were injected subcutaneously into the upper back of the mice. The width and length of tumors were measured by electronic digital display vernier caliper. Six weeks later, the mice were killed and tumors were removed for examination. The investigator was blinded to the group allocation during the experiment. The animal experiments were authorized by the Ethics Committee of Medical Research and New Technology, Shengjing Hospital of China Medical University (Ref#2016PS449K).

### Statistical analysis

Data were calculated as mean ± SD (or SEM) and analyzed by SPSS 19.0 (SPSS Inc., Chicago, USA). Student’s *t*-test was used for comparison between two groups. Pearson’s correlation analysis was used for correlations. Chi-square test was applied to assess the correlation between hsa_circRNA_0088036 expression and clinicopathologicaI characteristics in BC. Survival curves were determined by Kaplan-Meier method and compared using log-rank test. *P* < 0.05 was considered statistically significant.

## Supplementary information

Supplementary Material 1

Supplementary Material-Original Data

International Science Editing letter

Reproducibility checklist

Supplementary figure

Extended Data 1
