# Supplementary figures and images for: Hsa_circRNA_0088036 acts as a ceRNA to promote bladder cancer progression by sponging miR-140-3p
Source: Cell Death Dis. 2022 Apr 8;13(4):322. doi: 10.1038/s41419-022-04732-w (PMC8993833; doi:10.1038/s41419-022-04732-w)

— — — —

— — — —

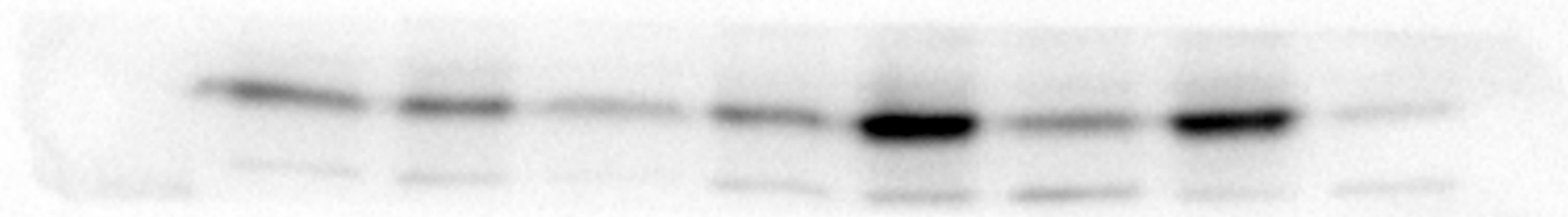

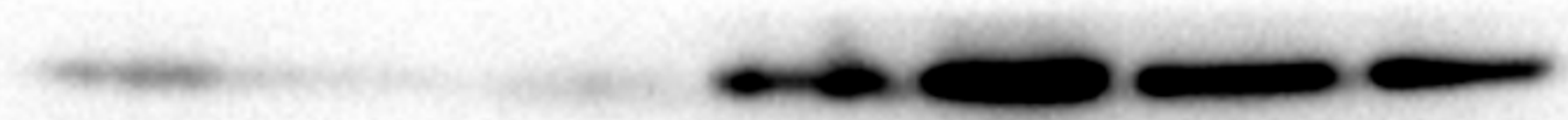

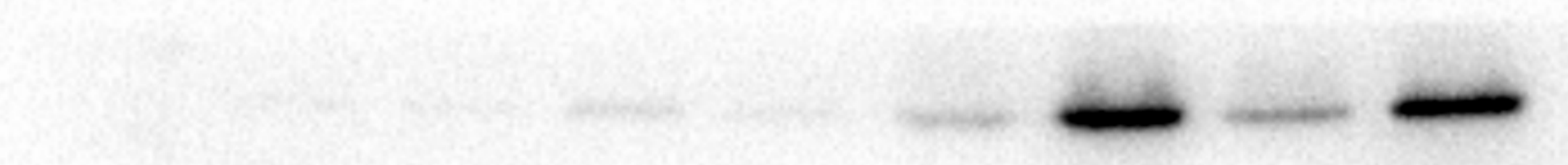

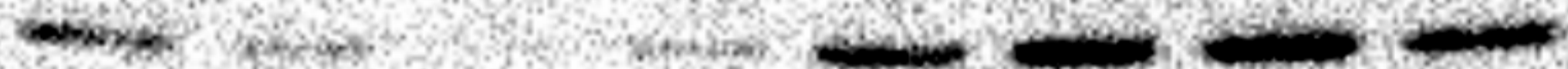

— — — —

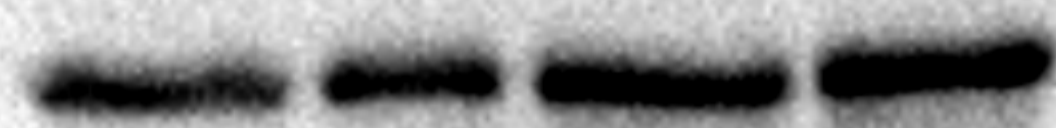

— — — —

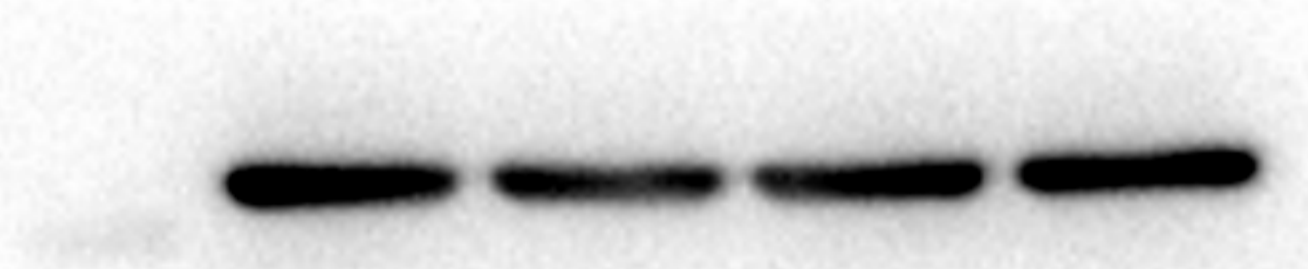

Supplement: Supplementary file 2 — Supplementary Material-Original Data [file 41419_2022_4732_MOESM2_ESM.pdf]
