# Supplementary material for: Hsa_circRNA_0088036 acts as a ceRNA to promote bladder cancer progression by sponging miR-140-3p
Source: Cell Death Dis. 2022 Apr 8;13(4):322. doi: 10.1038/s41419-022-04732-w (PMC8993833; doi:10.1038/s41419-022-04732-w)
Supplement: Supplementary file 3 — International Science Editing letter [file 41419_2022_4732_MOESM3_ESM.pdf]

International Science Editing

www.internationalscienceediting.com

Compuscript Ltd
T/A International Science Editing
Bay K, Shannon Industrial Park West
Shannon, Co Clare
Ireland
Tel. +353 61 472818 Fax +353 61 472688

DATE: June 8, 2021

To whom it may concern,

The paper "Hsa_circRNA_0088036 acts as a ceRNA to promote bladder cancer progression by sponging miR-140-3p" by Kefeng Wang was edited by International Science Editing. We were asked not to check the materials and methods, and references. Please contact us if you would like to view the edited paper.

Kindest regards,

David Cushley
