# Supplementary material for: Hsa_circRNA_0088036 acts as a ceRNA to promote bladder cancer progression by sponging miR-140-3p
Source: Cell Death Dis. 2022 Apr 8;13(4):322. doi: 10.1038/s41419-022-04732-w (PMC8993833; doi:10.1038/s41419-022-04732-w)
Supplement: Supplementary file 5 — Supplementary figure [file 41419_2022_4732_MOESM5_ESM.docx]

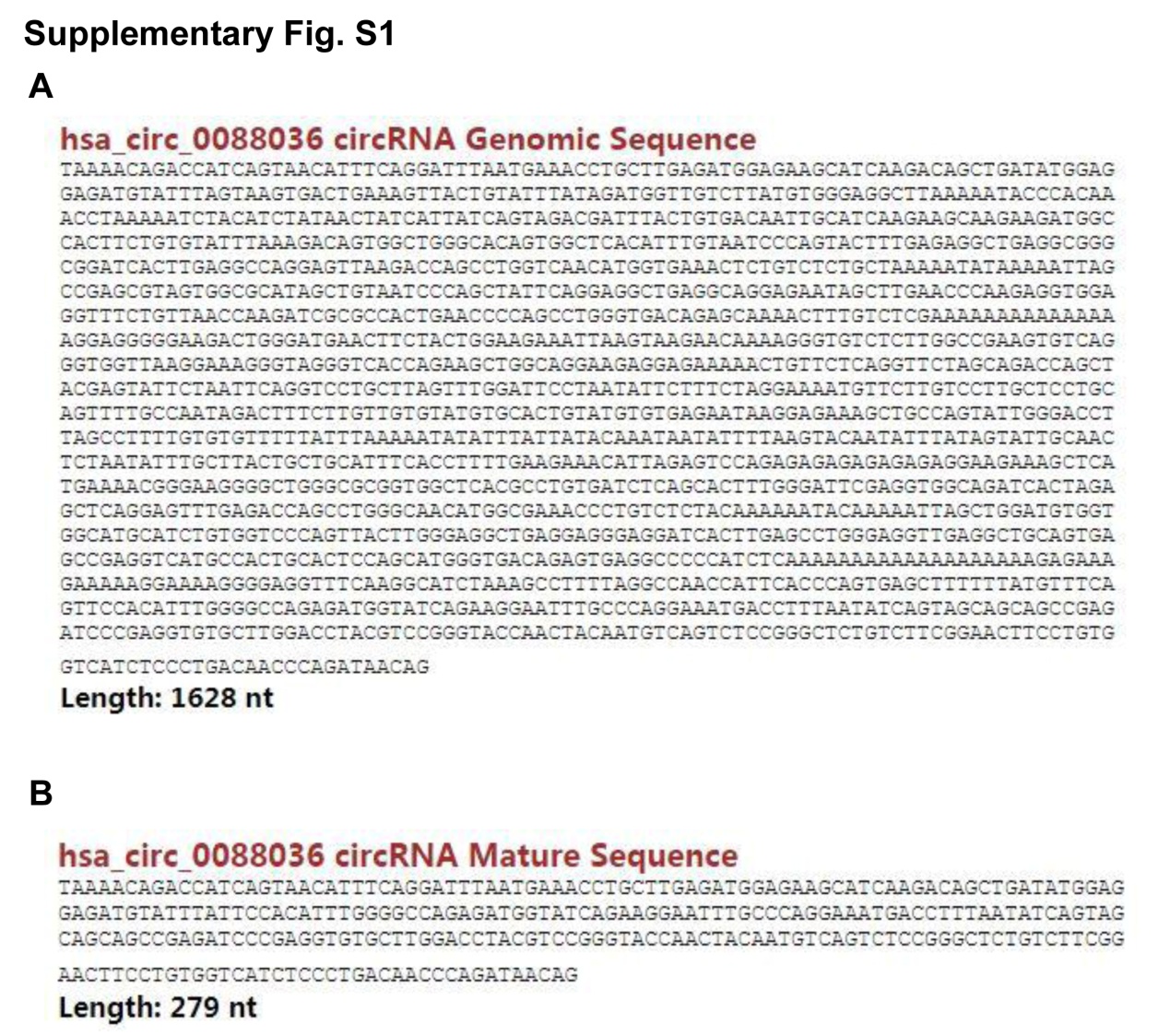


**Supplementary Fig. S1. (A)** The genomic length of hsa_circRNA_0088036. **(B)** The spliced sequence length of hsa_circRNA_0088036.
